# Age-Specific Excretion of Calcium, Oxalate, Citrate, and Glycosaminoglycans and Their Ratios in Healthy Children and Children with Urolithiasis

**DOI:** 10.3390/biom11050758

**Published:** 2021-05-19

**Authors:** Daniel Turudic, Anja Tea Golubic, Mila Lovric, Marko Bilic, Danko Milosevic

**Affiliations:** 1 Department of Pediatrics, University Hospital Centre Zagreb, 10000 Zagreb, Croatia; 2 Department of Nuclear Medicine and Radiation Protection, University Hospital Centre Zagreb, 10000 Zagreb, Croatia; anja.tea.golubic@gmail.com; 3 Department of Laboratory Diagnostics, University Hospital Centre Zagreb, 10000 Zagreb, Croatia; mlovric@kbc-zagreb.hr; 4 Department of Urology, University Hospital “Sveti Duh”, 10000 Zagreb, Croatia; mbilic1407@gmail.com; 5 School of Medicine, University of Zagreb, 10000 Zagreb, Croatia; danko.milosevic@zg.t-com.hr; 6 General Hospital Zabok and Croatian Veterans Hospital, 49210 Zabok, Croatia

**Keywords:** calcium, oxalate, citrate, glycosaminoglycans, urolithiasis, children

## Abstract

We analyzed children with urolithiasis with age- and gender-matched healthy children. Calcium (mmol/mmol creatinine) and the calcium/citrate ratio (mol/mmol) are the only variables that differentiate children before puberty from healthy children (ROC analysis confirmed only calcium/citrate as a significant variable with cut-off value > 0.84). Peri-pubertal children are distinguished from age- and gender-matched healthy children by the following variables: citrate (mmol/mol creatinine), calcium/citrate (mol/mmol), oxalate/glycosaminoglycans (mmol/g), oxalate/citrate ratios (mmol/mmol) and oxalate/(citrate × glycosaminoglycans) (mol oxalate × mol creatinine)/(mol citrate × g glycosaminoglycans). All variables were confirmed by ROC analysis with cut-off values ≤ 327.87, >1.02, >11.24, >0.12 and >0.03, respectively. These results indicate a different risk of urinary stones development before puberty vs. pubertal/postpubertal children and increasing importance (deficiency) of citrate and glycosaminoglycans in such children. J48 classifier confirmed the importance of the oxalate/(citrate × glycosaminoglycans) and the calcium/citrate ratios (Ox/Cit × GAG 0.22 and Cit/GAG 0.612) with the practically applicable classification tree for distinguishing between pubertal/postpubertal children with urolithiasis with age- and gender-matched healthy children.

## 1. Introduction

Ion interactions of urinary calcium (Ca), oxalate (Ox), and low urine citrate (Cit) may result in stone formation. Due to the strong affinity between Ca and Ox and the low calcium oxalate (CaOx) solubility product, hypercalciuria and hyperoxaluria are the most common cause of renal stones. The excretion of urinary citrate (Cit), which forms a soluble compound with Ca, reduces CaOx formation by depreciating the available Ca ions to interact with Ox [1,2,3,4,5,6,7,8]. Glycosaminoglycans (GAGs) (chondroitin sulfate, heparan sulfate, hyaluronic acid, dermatan sulfate, keratan sulfate) can also inhibit CaOx crystallization (this ability is attributed to at least the first three above) [1,9,10,11,12,13,14,15,16,17,18,19,20]. This study examines the relationship between urinary promoters/inhibitors and their ratios in children according to age. We consider GAGs’ separation into its constituents costly and, for the current pupose, impractical for everyday clinical practice.

## 2. Materials and Methods

### 2.1. Urine Sampling and Analysis

The study was conceived as an extension to our previous work of retrospective analysis of urinary stone disease in children from different parts of Croatia who had been treated for at least one urinary stone occurrence [20]. An ordinary abdominal X-ray (in selected cases, CT or MRI urography) and/or ultrasound were used for diagnosis. Children with glomerular diseases, urological anomalies, and coagulopathy were excluded from the study alongside hematuria of unknown origin. Children with acute urinary tract infections and metabolic abnormalities influencing urinary stone structure (i.e., children with hypercalciuria, hyperoxaluria, renal tubular acidosis, systemic metabolic diseases, hypokalemia, cystinuria, mucopolysaccharidoses) were excluded from the study too. Hypercalciuria was defined as urinary calcium excretion > 0.1 mmol/kg/24 h, primary hyperoxaluria as urinary oxalate excretion > 1 mmol/1.73 m^2^/24 h with glycolate excretion > 0.50 mmol/1.73 m^2^/24 h or l-glyceric acid excretion > 5 μmol/L, idiopathic or mild hyperoxaluria as oxalate excretion > 0.50 mmol/1.73 m^2^/24 h with normal glycolate/l-glyceric acid excretion for both inherited and acquired disorders. 

Primary hyperoxalurias (I, II, and III) and patients with possible nephrological metabolic/tubular disease were excluded by genetic testing [21,22,23]. Children with hypocitraturia (citrate excretion < 1.9 mmol/1.73 m^2^ for boys or <1.6 mmol/1.73 m^2^ for girls) with genetic/metabolic causes were excluded as well [24]. Our aim was to avoid the influence of any major promoters/inhibitors and to determine the possible effect of GAGs on the origin of stone formation. Enteric hyperoxaluria-causing diseases (i.e., celiac disease, cystic fibrosis, children with steatorrhea) were clinically excluded. The largest number of stones was obtained by spontaneous stone passage followed by extracorporeal shock-wave lithotripsy (ESWL) or endoscopic removal procedure, respectively, rarely by open surgery. CaOx urolithiasis in children was confirmed by infrared spectroscopy, and only these children were enrolled in the study. Gender, age, and urinary excretion of Ca, Ox, Cit, and GAGs were used for the study. In total, 25 healthy gender- and age-matched children with informed parental consent served as a control group. All children were enrolled in the study with informed parents/guardians and older children’s consent. For the urine samples to reflect a natural nutrient and fluid intake, the children were on a free diet. For the measurement of Ca, Ox, Cit and creatinine, 24-h urine collection was performed three days in a row. We used 24-h samples instead of 2-h morning or 12-h urine samples to acquire Ca, Ox, Cit, GAGs and creatinine excreted in urine as accurately as possible [25]. The intra-class correlation coefficient, built upon a trialed variable, was proven acceptable for further analysis (in the range of 0.79 to 0.93). 

A wide-mouth plastic bottle encompasses 10 mL of 6 N hydrochloric acid (preservative) was used for Ox, Cit and corresponding creatinine urine collection. The 24-h urine samples without the addition of hydrochloric acid were used for measuring GAGs and corresponding creatinine (cr) in urine [26]. The Combur 9 test (Boehringer Mannheim, Germany) was used as a nitrite marker as well as urine culture to rule out urinary infection. Calcium was measured by the cresolphthalein-complexone method [26]. Dionex Series 4000i gradient ion chromatography was used for Ox and Cit measurement and carbazole method for GAGs determination. Ca, Ox, Cit, and all promoter/inhibitor values were calculated with respect to cr. Such a calculation ensures that the urine is collected correctly and correction of the values to the same indicator [26,27].

Imprecision within batch for normal/high level (L1/L2) were for urine calcium from 0.51 to 0.56% and urine creatinine 0.45 to 1.15%. Imprecisions between batches were for urine calcium 0.815–1.04% and urine creatinine 0.475–1.11%. Accuracy determined by the recovery test for both levels was for calcium 98.5–103.5% and 98.4–103.5% for creatinine in urine. The method/analyte is included in the external proficiency testing scheme Instand.

Imprecision within batch for low/normal level (L1/L2) were for oxalate 2.45/0.42%, citrate 4.89/0.65%. Imprecision between the batch was for oxalate 7.01/5.51%, citrate 9.64/5.29%. Accuracy determined by the recovery test for all analytes was 98.5–103.5% for the normal level and 90.7–112.6% for low control levels. The method/analyte is included in the external proficiency testing scheme Reference Institute for Bioanalytics (Rfb).

### 2.2. Data Analysis

Urine excretion of Ca (mmol/mmol creatinine), Ox (mmol/mol cr), Cit (mmol/mol cr), GAGs (mg/mmol cr), Ca/Cit (mol/mmol), Ox/GAGs (mmol/g), Ox/Cit (mmol/mmol), Ox/(Cit × GAGs) (mol Ox × mol cr)/(mol Cit × g GAGs), and Cit/GAGs (mmol/g) were analyzed. Data are expressed as mean, standard deviation, median and interquartile range (IQR). The difference between subgroups was analyzed using the Mann–Whitney U-test [28]. Differences between multiple subgroups were analyzed using the Kruskal–Wallis test with Bonferroni multiple comparison [28]. These tests were selected due to their insensitivity to outliers and extremes because of the considerable dispersion of trialed variable data. Therefore, outliers and extremes are included in our analysis instead of the previously allowed homogenizing data practice by removing data outside the interval defined as the mean ± 2 standard deviations [29]. All applied tests were two-tailed, and *p*-values ≤ 0.05 were acknowledged as statistically significant. Practical significances of selected variables were analyzed using ROC analysis. Data analysis was performed by using Statistica for Windows version 8 (StatSoft, Dell, Inc.) and GraphPad Prism version 5 GraphPad, San Diego, CA, USA). Additionally, the J48 classifier was used to construct a classification model for discrimination between subgroups [30,31,32]. J48 is a useful supplement to ROC analysis because of its reduced sensitivity to any imbalance in group size and the overall size of groups.

## 3. Results

The study includes 61 children with proven CaOx urolithiasis with a 59 single and 2 recurrent stone episode. Only children with idiopathic urolithiasis were included in the study. Approximately 1/3 of the cases had a positive family history of urolithiasis. This group of children has compared with a group of 25 healthy children. Both groups are age- and gender-matched (gender differences; χ^2^-test, *p* = 0.906). Children with urolithiasis and the control group of healthy children were compared by age, Ca, Ox, Cit, GAGs, Ca/Cit, Ox/Cit, Ox/GAGs, Cit/GAGs and Ox/Cit × GAGs variables.

Groups of healthy children and children with urolithiasis were divided into subgroups according to age median criteria (expressed in months, mo.). Healthy children are therefore divided into 2 separate subgroups; a subgroup of younger children (YC) (*n* = 13) and a subgroup of older children (OC) (*n* = 12). Accordingly, children with urolithiasis also formed 2 subgroups, YC (*n* = 31) and OC (*n* = 30), using the median for healthy children. The age median of children was determined for individual YC and OC subgroups. Differences between healthy children vs. children with urolithiasis of both YC and OC subgroups are analyzed. Ca and Ca/Cit are the only variables differentiating YC subgroups with significantly higher values for children with urolithiasis. OC subgroups had significantly higher values in healthy children for the Cit, Ca/Cit, Ox/GAG, Ox/Cit, Ox/(Cit × GAG) variables (Table 1 and Table 2).

**Figure 1:**
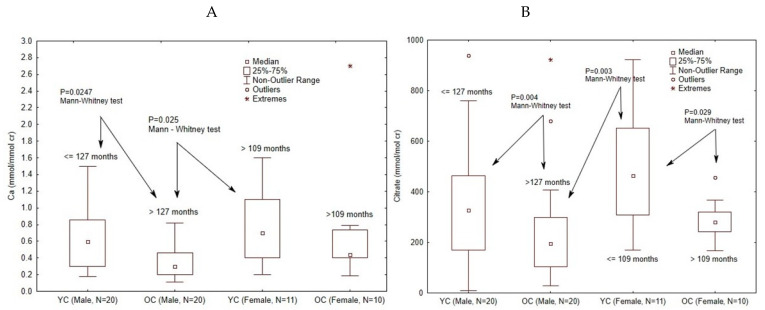
Possible gender differences between subgroups were tested by the Kruskal–Wallis test and Bonferroni multiple comparisons. Differences between female YC vs. OC male groups (*p* = 0.008) were further test by post-hoc Mann–Whitney U test in accordance with cut-off values to our gender median (female 109 mo. and male 127 mo.). Significant differences were found for Ca (female YC vs. male OC and male YC vs. male OC) in section (**A**) and Cit excretion (female YC vs. male OC, female YC vs. female OC, and male YC vs. male OC) in section (**B**).

Clinical staging of puberty was not a part of this study from the beginning of study. However, as we found possible gender differences between subgroups, these were tested by the Kruskal–Wallis test and Bonferroni multiple comparisons. A significant difference was found for Ca and Cit between female YC vs. OC male groups (*p* = 0.008). These differences were further tested by post-hoc Mann–Whitney U test with our children’s median values for gender (female 109 mo. and 127 mo. for male children). We found significant differences in the variables mentioned above regarding excretion of Ca (female YC vs. male OC and male YC vs. male OC). Significant differences were found also for Cit excretion (female YC vs. male OC, female YC vs. female OC and male YC vs. male OC (Figure 1). Excretion of Ox was not statistically significant.

Our group of children was carefully selected to fully express promoter/inhibitor ratios and GAGs in adherence with inclusion/exclusion criteria. Therefore, they represent a relatively homogenized group by excluding all potent and already known promoters. The resulting cohort of children has no prominent stone-forming predictors. The drawback of such criteria are relatively small groups with limited possibility to form firm conclusions.

Therefore, the above-listed variables of children were analyzed using ROC analysis. Judging by their AUC and *p*-values that fit potential diagnostic abilities, variables are listed according to their *p*-values. The results of ROC analysis for statistically significant variables are shown in Table 3. Statistically significant differences were found between healthy children vs. children with urolithiasis for Cit, Ca/Cit, Ox/GAG, Ox/Cit and Ox/Cit × GAGs variables. Distinctive sensitivity with low specificity was found for all examined variables. A significant difference with high sensitivity was found in YC only for the Ca/Cit variable. OC show moderate/high sensitivity for Cit, Ca/Cit, Ox/GAG, Ox/Cit and Ox/Cit × GAGs variables but with low to moderate specificity. All variables have a significant number of outliers and extremes. A Cit variable was found low in OC with urolithiasis vs. healthy children. At the same time, Ca/Cit, Ox/Cit and GAGs ratios (Ox/GAGs and Ox/Cit × GAGs) were significantly higher in OC with urolithiasis. Differences found in Ca, Ox, Cit and GAGs excretion in healthy children vs. children with urolithiasis were useful for estimation of Cit, Ox and GAGs post-puberty changes.

Some children due to their metabolic urinary values belong to the outliers/extremes. Urinary analysis, performed 3 times, revealed mild hyperoxaluria in two children marked as extremes as well as two children with hypocitraturia. The same applies to one child with the lowest oxalate/>0.1 mmol/kg value x ratio of GAG (outlier) who has a GAG significantly below the values of healthy children.

**Figure 2:**
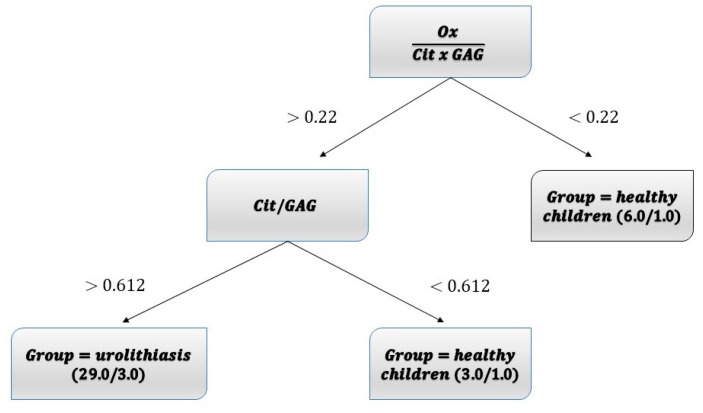
Classification tree for discrimination between OC healthy children and children with urolithiasis. The classification tree has two decision nodes and three decision leaves (for clarity, all variables are designated by full names instead by abbreviations, except for glycosaminoglycans (GAG).

To optimally classify healthy children vs. children with urolithiasis J48 classifier was applied (Figure 2). Only healthy OC vs. OC with urolithiasis are successfully classified. J48 classifier uses Ox/Cit × GAG and Ca/Cit ratios to discriminate between OC with urolithiasis from OC healthy children. The classification tree has two decision nodes and three decision leaves (Figure 2 and Table 4).

DECISION NODE 1

IF Ox/(Cit × GAG) ratio < 0.22 THEN group = healthy children (precision 6.0/1.0)

ELSE IF Ox(Cit × GAG) ratio ≥ 0.22 THEN DECISION NODE 2

 

DECISION NODE 2

IF Ca/Cit ratio < 0.612 THEN group = healthy children (precision 3.0/1.0)

ELSE IF Ca/Cit ratio ≥ 0.612 THEN group = urolithiasis (precision 29.0/3.0)

The number of correctly classified children is 38 of 42, i.e., 90.8%, with Kappa statistics of 0.754 (Table 4)

## 4. Discussion

Children develop physically and mentally over time. This is a period marked with numerous changes in the endocrine and metabolic regulations, of which puberty is one of the most important. We, therefore, decided to evaluate the median age as a distinguishing factor, primarily because it may coincide with peri-puberty in children. The possibility of changing interrelation of Ca, Ox, Cit and GAGs excretion during the peri-pubertal period motivated us to investigate the possible changing risk of urolithiasis in this developmental period. We divided the study children into young and older groups in order to investigate the changing risk of urolithiasis during this period. Cut-off values for the timing of puberty (female 125 mo. and male 133 mo.) were considered as referral values [33]. We believe that a differences found in Ca and Cit between female YC vs. OC male groups as well as for Cit female YC vs. OC and male YC vs. OC indicating peri-pubertal changes. It is likely that peri-pubertal changes account for the observed differences in the profiles for calcium, citrate and GAG excretion of young and older children

Studies analyzing urinary promoter/inhibitor variables such as Ca, Ox, Cit and their ratios in children are uncommon in literature. Since Ca is a strong promoter of urolithiasis with possible masking influences over the other promoters/inhibitors, we assumed that previous researchers’ negative results of GAGs excretion in children’s urine with urolithiasis might be partly due to the inclusion of participants with prominent metabolic promoters (hypercalciuria included) [16,26]. Previous researchers of Ox, Cit and GAGs interactions frequently opted for Ca exclusion [10,11]. We also found Ca excretion in urine as an important contributor to urinary stone development in children with urolithiasis, significantly higher in female YC vs. male OC. A higher Ca excretion was found in YC in comparison with corresponding male OC. At the same time, the Ca/Cit ratio shows substantial differences in all children, YC and OC alike, between healthy children and children with urolithiasis. Even though Ca excretion was moderately significant (*p* < 0.05) only in YC (not confirmed by ROC analysis), we can assume that increased urinary Ca excretion with the given excretion of Cit is the most plausible explanation for the higher risk of stone formation in YC with urolithiasis. Therefore, in the young children, the raised Ca/Cit and increased risk for stones was due to increased Ca excretion and not to a decrease in Cit.

Figure 1 clearly shows that the ratios of Ca/cr and Cit/cr were significantly higher in young boys compared with older boys, and similarly for girls. This is probably largely attributable to use of creatinine as denominator in the calculation. In healthy children there is a progressive linear increase in creatinine excretion from low values in early life to peak in late adolescence which is due to increasing skeletal muscle. There is little difference between sexes in young children, but there is divergence from puberty with higher creatinine excretion in boys [34,35].

Urinary oxalate excretions were previously investigated in Croatia in children with genetic diseases (primary hyperoxalurias I, II, III) [26,27,36,37]. As high oxalate in urine decreases the inhibitory effectiveness and protection from crystallization, we opted for primary hyperoxalurias exclusion from all calculations [13].

The age difference in stone risk formation and a decrease in Cit excretion was previously described in the literature [6,38,39]. It has already been reported that besides hypercalciuria, hypocitraturia is the most common association with urolithiasis [3,4,5,6,7,8]. We observed a decrease in Cit excretion of healthy male and female OC, with higher Cit reduction in males in comparison with females, both indicating postpuberty Cit excretion decrease. The highest fall of Cit excretion was found between female YC vs. male OC. Lower Cit excretion in OC is the main cause for higher values of Ox/Cit and Ox/Cit × GAGs ratios in children with urolithiasis. The importance of urine Cit excretion is shown in the decision tree model using the Ca/Cit ratio as the second decision node in OC. Interaction of inhibition of the NaDC-1 citrate transporter by the oxalate transporter slc26a6 in the absence of hypercalciuria and/or calcium sensor receptor alteration may be an answer to the question of whether stone-free or stone-recurrence in OC will occur and explain some of our outliers and extremes [40,41,42].

Articles detailing macromolecules, such as the excretion of GAGs and their urine ratios, are exceedingly rare. A definitive conclusion of their interactions (depletion) with other promoters/inhibitors of crystallization is still not possible to define. An increase of GAGs excretion in infants was previously observed [2]. As promoter/inhibitor ratios (Ox/GAGs and Ox/Cit × GAGs) isolate stone-forming OC and healthy group, the only plausible explanation is influence of GAGs on stone formation in infants. Therefore, due to a lower GAGs urine excretion in OC, Ox/GAGs and Ox/Cit × GAG ratios are higher in children with urolithiasis compared with healthy children. Ox/Cit × GAG ratio combined with Ca/Cit ratio distinguishes OC children with urolithiasis from healthy children at the first decision node. Statistical significant Ox/Cit × GAG ratio in OC indicates a decrease in GAG excretion of postpubertal children. Although we excluded all known genetic and metabolic diseases, there is a possibility of yet undiscovered genetic and metabolic disruptors in our stone formers. The model to pinpoint candidate(s) for unknown genetic mutations and diseases, is to search for outliers and extremes in a meticulously controlled study. In our stone formers, there are some possible candidates for such diseases. They include children with mild hyperoxaluria and oxalate excretion between >500 and <1000 µmol/1.73 m^2^/24-h body weight of yet unknown origin, children with hypocitraturia and a child with urinary GAGs considerably lower than normal children. Most outliers and extremes have all three components of Ox/Cit × GAG ratio close to upper (oxalate) or lower (citrate and GAGs) normal limits.

Lithogenic protection in children’s urine is higher compared with adults due to a higher excretion of macromolecules, including increased excretion of Cit, GAG and fibronectin [2,4,43,44]. Elevated GAGs excretion in infants and toddlers’ urine is explained by increased excretion of proteoglycans in longitudinally growing bones [43]. This protective role of GAGs is consistent with observation for the lowest incidence of urinary stone formation in children 0–3 years [45]. Articles describing excretion of GAGs in urine are scarce and with no clear consensus [16,42,43,44,46]. Reduced excretion of GAGs’ in urine is rarely reported in children [15,42,44,46]. Although some constituents of GAGs have an inhibitory role, others could even promote CaOx crystallization [17,42,46]. The protective part of GAGs depends on their degree of sulfation, i.e., the number of anionic charged sites on the surface [14,17,47]. This protection is also achieved by inhibition of CaOx monohydrate crystal adherence to renal epithelial cells by GAGs [9,18,19,47]. GAGs constituents (chondroitin sulfate and heparan sulfate) may act as a polymer with an aggregative effect, promoting small crystal forming but inhibiting grand crystal growth [42,46,47]. Increased GAGs production induced by tubular injury in the presence of CaOx crystals and Ox ions is well documented, as well as GAGs’ role in reducing renal tubular cell injury caused by crystals and their oxidative stress-induced apoptosis [2,9,14,44]. The fact that GAGs depletion in urine is found in some studies and by other unaltered, compared to healthy subjects, may reflect GAGs’ adequate or inadequate activity to suppress tubular injury when the samples were actually taken for analysis [15,16]. Therefore, research of urinary stone formation overseen immediately or after a considerable time since acute stone episode may contribute to different results. Pathological values of urinary GAGs in stone disease of children are undefined, as is the possibility of mutation leading to urine GAG depletion [4,46]. To our knowledge, any attempt to identify GAG’s only defect in urinary stone disease has not yet succeeded.

GAGs are better expressed, and their inhibitory potential is evaluated as part of ratios with Ox and Cit [10,11,12]. In Ox/Cit × GAG ratio, we assessed urinary Ox known to have the stone promoting capability as a counter with two inhibitors as denominators [26,27]. We noticed that all three ratio components could be within normal ranges, and still, the outcome of the ratio reaches end-cumulative pathological values. The result may get significant differences for stone formers in comparison to healthy children. Amplifying minor differences in urine composition of this ratio’s components was already observed [10,26,27]. Low urinary Cit and consequential GAGs depletion can explain the positive result of Ox/Cit, Ox/GAG andOx/Cit × GAGs ratio. Some ratio disturbances can be corrected because Cit intake increases GAG excretion in the urine, but the opposite vice versa can also be assumed [1]. The constant decline in Cit and GAG values during aging in OC can affect the stone’s promotion more than previously expected. It remains to be determined whether this shift in promoter/inhibitor disturbance is due to some peri-puberty hormonal changes. The post-pubertal excretion values of Ox, Cit and GAG in OC appear to match the risk of adult urinary stones better. Therefore, in absence of hypercalciuria and primary hyperoxalurias in OC a cumulative effect of Ox, Cit and GAGs disturbance(s) with relatively slow growth of urinary stone is assumed, probably similar to stone genesis in adults.

## Figures and Tables

**Table 1:** Descriptive statistics and Mann–Whitney U-test for YC with urolithiasis and matched control group of healthy children (M = male, F = female).

Total No. of Children	Healthy Children (*n* = 25)		Children with Urolithiasis (*n* = 61)		*p* Value
Variable	Healthy Children (YC) (*n* = 13) M:F = 5:8		YC Children with Urolithiasis (*n* = 31), M:F = 18:13 31 Single Stone Occurrence, 0 Recurrent Family History Positive (11 Total, 35.4%)		
	Mean (SD)	Median (IQR)	Mean (SD)	Median (IQR)	
Age (months)	66.23 (39.07)	80.00 (40.00 to 90.00)	75.71 (31.64)	84.00 (58.00 to 103.00)	0.3679
Ca (mmol/mmol cr)	0.57 (0.74)	0.20 (0.19 to 0.56)	0.66 (0.40)	0.60 (0.34 to 0.99)	0.0421
Ox (mmol/mol cr)	111.58 (84.02)	79.21 (64.40 to 122.20)	72.44 (49.07)	62.63 (43.00 to 84.74)	0.0971
Cit (mmol/mol cr)	438.49 (279.36)	368.10 (337.00 to 408.80)	391.36 (232.55)	380.42 (219.00 to 507.20)	0.7674
GAG (mg/mmol cr)	5.40 (3.60)	4.25 (3.93 to 5.40)	4.80 (3.78)	3.54 (2.15 to 6.20)	0.3347
Ca/Cit	1.06 (0.87)	0.84 (0.94 to 1.39)	4.54 (12.18)	1.60 (0.97 to 3.27)	0.0140
Ox/GAG	25.63 (22.36)	18.64 (14.21 to 22.95)	22.35 (15.09)	22.20 (10.13 to 39.76)	0.8672
Ox/Cit	0.26 (0.14)	0.22 (0.19 to 0.34)	0.39 (0.73)	0.18 (0.11 to 0.29)	0.2418
Ox/(Cit × GAG)	0.06 (0.05)	0.05 (0.04 to 0.07)	0.16 (0.50)	0.05 (0.03 to 0.10)	0.9692
Cit/GAG	109.93 (99.32)	85.18 (56.08 to 95.34)	127.62 (120.39)	84.66 (47.10 to 160.47)	0.8269
Cr (mmol/day)	3.30 (1.80)	3.86 (1.56 to 4.96)	4.31 (2.42)	3.90 (3.10 to 5.02)	0.2687

**Table 2:** Descriptive statistics and Mann–Whitney U-test for OC with urolithiasis and matched control group of healthy children (M = male, F = female).

Total No. of Children	Healthy Children (*n* = 25)		Children with Urolithiasis (*n* = 61)		*p* Value
Variable	Healthy Children (OC) (*n* = 12) M:F = 6:6		OC Children with Urolithiasis (*n* = 30), M:F = 22:8 28 Single Stone Occurrence, 2 Recurrent Family History Positive (11 Total, 36.67%)		
	Mean (SD)	Median (IQR)	Mean (SD)	Median (IQR)	
Age (months)	142.67 (26.31)	134.50 (122.50 to 157.00)	157.13 (25.49)	158.50 (133.00 to 176.00)	0.0818
Ca (mmol/mmol cr)	0.34 (0.17)	0.36 (0.19 to 0.49)	0.46 (0.47)	0.33 (0.25 to 0.59)	0.6065
Ox (mmol/mol cr)	44.94 (21.60)	44.15 (27.15 to 56.32)	67.52 (43.91)	53.54 (34.81 to 86.74)	0.1157
Cit (mmol/mol cr)	363.89 (152.65)	361.71 (276.74 to 414.08)	261.59 (184.41)	239.61 (126.89 to 321.12)	0.0154
GAG (mg/mmol cr)	3.66 (2.38)	3.54 (1.51 to 4.82)	2.42 (1.93)	1.78 (1.24 to 3.00)	0.0794
Ca/Cit	1.06 (0.77)	0.85 (0.54 to 1.41)	2.26 (2.01)	1.50 (1.10 to 2.63)	0.0113
Ox/GAG	22.23 (35.33)	10.46 (7.00 to 31.34)	39.47 (37.50)	27.74 (16.09 to 44.07)	0.0278
Ox/Cit	0.14 (0.09)	0.11 (0.09 to 0.18)	0.39 (0.46)	0.25 (0.14 to 0.46)	0.0113
Ox/(Cit × GAG)	0.08 (0.09)	0.02 (0.02 to 0.14)	0.24 (0.29)	0.11 (0.05 to 0.30)	0.0058
Cit/GAG	136.61 (89.87)	98.55 (82.35 to 191.29)	152.32 (110.21)	133.04 (66.36 to 209.49)	0.8237
Cr (mmol/day)	8.10 (3.28)	7.85 (5.96 to 10.60)	8.14 (4.30)	7.27 (5.56 to 10.30)	0.8021

**Table 3:** Summary of ROC analysis for statistically significant variables.

Healthy YC Children (*n* = 13) vs. YC with Urolithiasis (*n* = 31)					
Variables	Cut-off value	AUC	Sensitivity	Specificity	*p*-value
Ca/Cit	>0.84	0.737 (0.571 to 0.903)	83.87	53.85	0.0050
**Healthy OC Children (*n* = 12) vs. OC with Urolithiasis (*n* = 30)**					
Variables	Cut-off value	AUC	Sensitivity	Specificity	*p*-value
Cit (mmol/mol cr)	≤327.87	0.742 (0.578 to 0.906)	80.00	66.67	0.0039
Ca/Cit	>1.02	0.753 (0.585 to 0.921)	76.67	66.67	0.0032
Ox/GAG	>11.24	0.719 (0.515 to 0.924)	86.67	66.67	0.0355
Ox/Citrate	>0.12	0.753 (0.601 to 0.904)	80.00	75.00	0.0011
Ox/(Cit × GAG)	>0.03	0.775 (0.600 to 0.950)	93.33	58.33	0.0021

**Table 4:** The performance measures for classification tree (confusion matrix).

Class	J48 Classification	
Healthy children (*n* = 12)	Healthy children (*n* = 9)	Urolithiasis (*n* = 3)
Urolithiasis (*n* = 30)	Healthy children (*n* = 1)	Urolithiasis (*n* = 29)
